# Epigallocatechin gallate inhibits release of extracellular vesicles from platelets without inhibiting phosphatidylserine exposure

**DOI:** 10.1038/s41598-021-97212-8

**Published:** 2021-09-03

**Authors:** Sarah L. Millington-Burgess, Matthew T. Harper

**Affiliations:** Department of Pharmacology, University of Cambridge, Tennis Court Road, Cambridge, CB2 1PD UK

**Keywords:** Mechanism of action, Cardiovascular diseases

## Abstract

Arterial thrombosis triggers myocardial infarction and is a leading cause of death worldwide. Procoagulant platelets, a subpopulation of activated platelets that expose phosphatidylserine (PS), promote coagulation and occlusive thrombosis. Procoagulant platelets may therefore be a therapeutic target. PS exposure in procoagulant platelets requires TMEM16F, a phospholipid scramblase. Epigallocatechin gallate (EGCG) has been reported to inhibit TMEM16F but this has been challenged. We investigated whether EGCG inhibits PS exposure in procoagulant platelets. PS exposure is often measured using fluorophore-conjugated annexin V. EGCG quenched annexin V-FITC fluorescence, which gives the appearance of inhibition of PS exposure. However, EGCG did not quench annexin V-APC fluorescence. Using this fluorophore, we show that EGCG does not inhibit annexin V binding to procoagulant platelets. We confirmed this by using NBD-labelled PS to monitor PS scrambling. EGCG did not quench NBD fluorescence and did not inhibit PS scrambling. Procoagulant platelets also release PS-exposing extracellular vesicles (EVs) that further propagate coagulation. Surprisingly, EGCG inhibited EV release. This inhibition required the gallate group of EGCG. In conclusion, EGCG does not inhibit PS exposure in procoagulant platelets but does inhibit the EV release. Future investigation of this inhibition may help us further understand how EVs are released by procoagulant platelets.

## Introduction

Arterial thrombosis, the formation of a blood clot inside an artery, is the major trigger for myocardial infarction and is a leading cause of death worldwide^1,2^. Platelets are key regulators of arterial thrombosis. Rupture of an atherosclerotic plaque leads to platelet adhesion and activation. Two phenotypes of activated platelet can be observed during thrombosis. ‘Pro-aggregatory’ platelets have activated integrin α_IIb_β_3_, allowing them to aggregate in a fibrinogen-dependent manner. This aggregation forms a large platelet plug that may occlude the arterial lumen. However, a subpopulation of activated platelets become ‘procoagulant’ platelets. These platelets downregulate active α_IIb_β_3_. Instead, these platelets expose phosphatidylserine (PS), which forms a binding site for the tenase and prothrombinase coagulation complexes, accelerating the activity of these complexes and protecting them from plasma-based inhibitors^3–7^. Procoagulant platelets therefore localise coagulation to the site of plaque rupture. Local thrombin generation is enhanced, leading to extensive fibrin formation that stabilises the platelet aggregate. Procoagulant platelets are therefore crucial to formation of a stable thrombus^8,9^. Procoagulant platelets also release PS-exposing extracellular vesicles (EVs) that further increase procoagulant activity^10^. Procoagulant platelets may therefore be a valuable therapeutic target.

PS exposure in procoagulant platelets is less well understood than the signalling that occurs in pro-aggregatory platelets, but some key events have been established. In resting platelets, PS is restricted to the inner leaflet of the plasma membrane by an ATP-dependent aminophospholipid translocase (known as the ‘flippase’). PS exposure is triggered by a very high, sustained increase in cytosolic Ca^2+^ concentration ([Ca^2+^]_cyt_)^11^. During physiological activation, this occurs downstream of the mitochondrial permeability transition pore^12,13^, although it can be artificially activated by Ca^2+^ ionophores. The high increase in [Ca^2+^]_cyt_ in procoagulant platelets triggers inactivation of this flippase and activation of a bi-directional phospholipid ‘scramblase’. The net result is exposure of PS in the outer leaflet of the plasma membrane. The scramblase has been identified as TMEM16F^14^. Platelets from *Tmem16f*^–/−^ mice have reduced PS exposure and reduced release of PS-exposing EVs^15–17^. Similarly, mutations in TMEM16F result in reduced platelet PS exposure and PS-exposing EV release in Scott Syndrome^18^. Inhibition of TMEM16F may therefore be an effective way to inhibit platelet procoagulant activity.

Epigallocatechin gallate (EGCG) has been reported to inhibit TMEM16F activity in multiple studies^19–21^. EGCG is the major catechin polyphenol in green tea and has been associated with a wide range of health benefits^22,23^. Although the low oral bioavailability of EGCG means that many of the benefits proposed on the basis of in vitro experiments are unlikely to be replicated in vivo^24,25^, EGCG and related catechins may be useful templates for drug design^26–30^. However, Le et al.^31^ recently reported that EGCG does not, in fact, inhibit murine TMEM16F expressed in HEK293 cells. Rather, they propose that EGCG quenches the fluorescence of many fluorophores used in previous studies, giving the appearance of inhibition of PS exposure. In is therefore important to establish whether EGCG inhibits PS exposure catalysed by endogenous TMEM16F in human platelets. In this study, we confirm that EGCG does not inhibit PS exposure in human platelets. Despite this, we find that EGCG inhibits the release of EVs from procoagulant platelets.

## Results

### EGCG appears to inhibit platelet PS exposure and release of PS-exposing EVs

**Figure 1:**
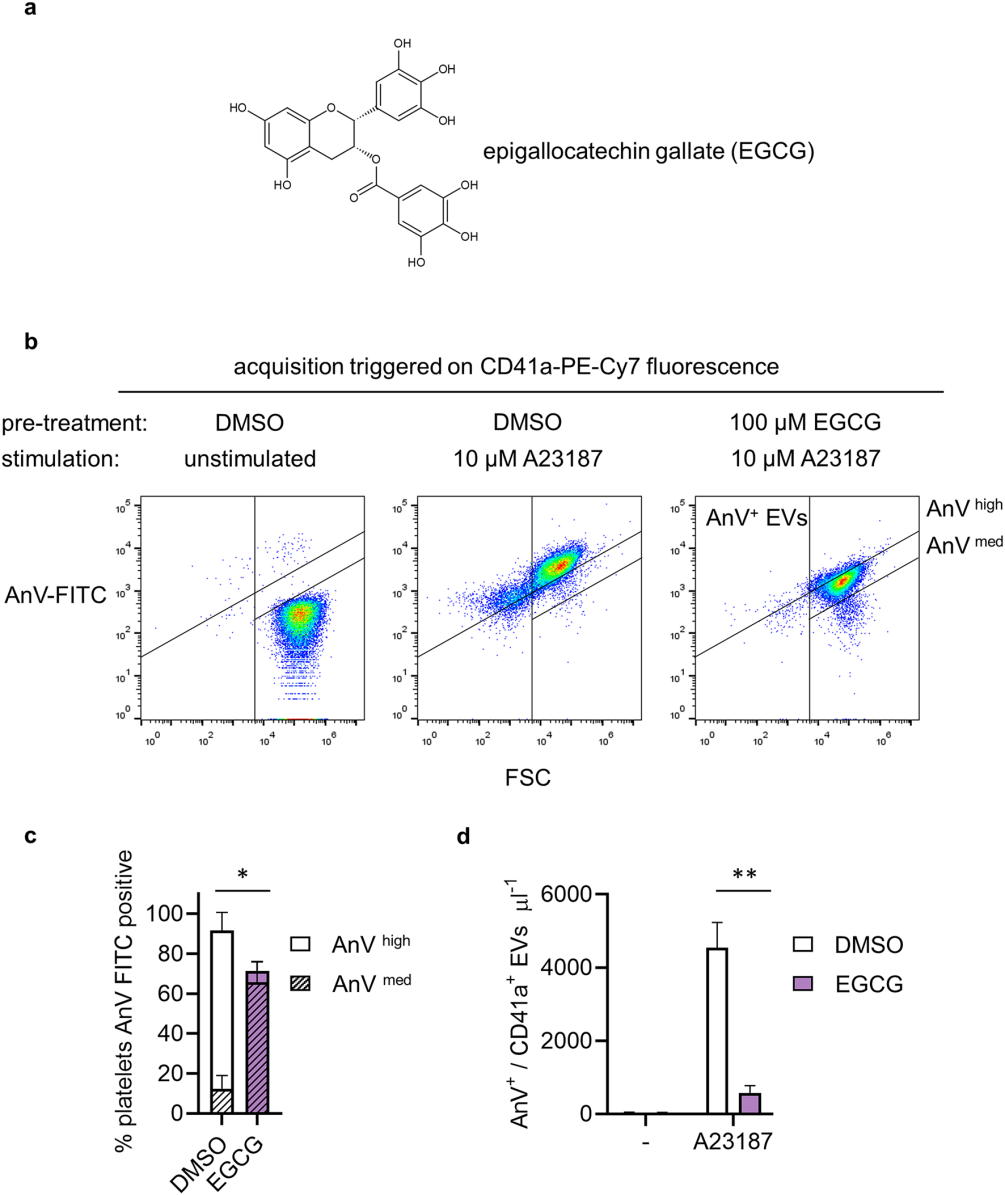
EGCG appears to inhibit platelet PS exposure and release of PS-exposing EVs. Washed platelets were treated with epigallocatechin gallate (EGCG; 100 μM; 10 min; structure shown in (**a**) or the vehicle (DMSO) as control, then stimulated with of A23187 (10 μM; 10 min). Samples were stained with anti-CD41a-PE-Cy7 to trigger fluorescence acquisition of CD41^+^ events. Annexin V (AnV)-FITC was used to detect PS exposure. The panels in (**b**) show density plots of events from low density (blue) to high density (red) of forward scatter (FSC-A) and FITC fluorescence. Density plots in this figure and subsequently were generated in FlowJo v10.7 (https://www.flowjo.com/). AnV^+^ platelets could be resolved into ‘high’ (AnV^high^) and ‘medium’ (AnV^med^) levels of fluorescence, as indicated. The vertical line separating left and right was defined in a previous study by the FSC-A of 1 µm silica beads^32^. The density plots are representative of data from 5 different donors. In (**c**), the percentage of platelets with high and medium levels of AnV-FITC fluorescence are shown. In (**d**), the number of AnV^+^/CD41a^+^ EVs per μl are shown. Data are presented as mean ± s.e.m. (n = 5). *p < 0.05; **p < 0.01.

To investigate the effect of EGCG on platelet procoagulant activity, washed platelets were treated with EGCG (100 μM; 10 min; Fig. 1a) or its vehicle (DMSO) as control, prior to stimulation with the Ca^2+^ ionophore, A23187 (10 μM; 10 min). A23187 was used rather than physiological agonists, such as thrombin and collagen, in order to bypass the potential of effects of EGCG on intracellular signalling immediately downstream of platelet receptors. Following stimulation, vehicle-treated platelets and released EVs were stained with anti-CD41a-PE-Cy7 and AnV-FITC and analysed by flow cytometry. PE-Cy7 fluorescence was used to trigger event acquisition, which allows smaller events to be resolved than when forward scatter is used to trigger event acquisition. As shown in Fig. 1, A23187 induced AnV-FITC binding to almost all platelets and induced the release of PS-exposing EVs, defined here as CD41a^+^/AnV^+^ events that were gated from platelets based on FSC/SSC profile. As discussed previously^32^, the number of events detected in this manner is likely to be an underestimate of the total number of PS-exposing EVs released by these platelets and is predominantly a measure of the largest EVs released.

Prior treatment with EGCG appeared to inhibit these responses. Platelet AnV-FITC fluorescence was significantly inhibited. Although the percentage of platelets binding AnV-FITC to some extent was not significantly reduced (Fig. 1b,c), the level of FITC fluorescence was lower in EGCG-treated platelets, such that AnV positive platelets could be resolved into high fluorescence (AnV ^high^) and medium fluorescence (AnV ^med^) populations, both with higher fluorescence than unstimulated platelets (Fig. 1b). Essentially, EGCG reduced most annexin V binding from AnV ^high^ to AnV ^med^ (Fig. 1c). Moreover, the number of PS-exposing EVs detected was also significantly inhibited (Fig. 1d).

These data initially suggested that EGCG may inhibit the release of PS-exposing EVs, possibly by inhibiting the extent of platelet PS exposure. However, Le et al. recently reported that EGCG may quench the fluorescence of some fluorophores, giving an alternative explanation for the apparent effect of EGCG^31^. We therefore sought to investigate whether EGCG really has any effect on PS exposure and release of PS-exposing EVs in platelets.

### EGCG and related catechin polyphenols quench AnV-FITC fluorescence but not AnV-APC

**Figure 2:**
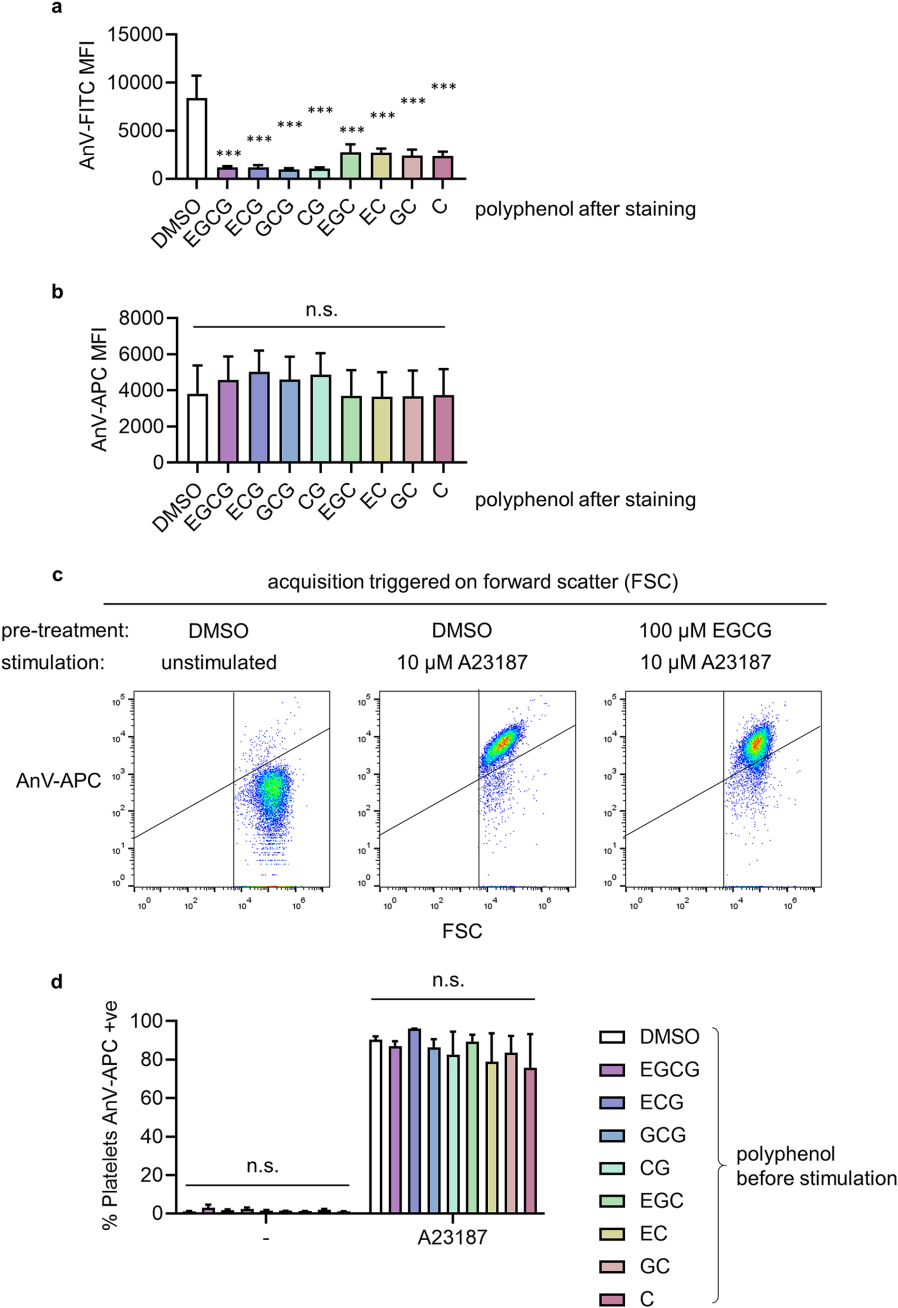
EGCG and related catechin polyphenols quench AnV-FITC fluorescence but not AnV-APC. Platelets were stimulated with A23187 then stained with AnV-FITC (**a**) or AnV-APC (**b**), fixed, then treated with the indicated catechin polyphenol (or DMSO as vehicle control). The median fluorescence intensity (MFI) is shown (mean ± s.e.m.; n = 3–4; ***p < 0.05; n.s. not significant). In (**c**) and d, platelets were treated with EGCG (or other catechin polyphenols in d) or DMSO before stimulation with A23187, as indicated. Samples were stained with AnV-APC; acquisition was triggered on forward scatter (FSC). Note that EVs are not readily resolved when acquisition is triggered on FSC. c shows representative density plots, and the percentage of AnV-APC positive platelets are quantified in (**d**) (mean ± s.e.; n = 3; n.s. = not significant). The catechin polyphenols are: epigallocatechin gallate (EGCG), epicatechin gallate (ECG), catechin gallate (CG), epicatechin (EC), catechin (C), gallocatechin gallate (GCG), epigallocatechin (EGC) and gallocatechin (GC).

To test whether EGCG directly affected AnV-FITC fluorescence, platelets were stimulated with A23187 to trigger PS exposure, then incubated with EGCG (20 μM) or vehicle control. EGCG significantly quenched AnV-FITC fluorescence (Fig. 2a). We therefore tested several related catechin polyphenols (see Supplementary Table 1 for structures), to determine whether any of these molecules did not quench AnV-FITC fluorescence. These polyphenols are based on a common catechin core but vary in the presence of an additional alcohol group in the gallocatechins and the presence of a gallate group (trihydroxybenzoic acid). Despite the variations, all these related catechin polyphenols also quenched AnV-FITC fluorescence. We therefore investigated whether other fluorophore conjugates of AnV might be more suitable. In contrast to AnV-FITC, the fluorescence of AnV-APC (detected on FL4) was not significantly affected by EGCG or the related catechin polyphenols (Fig. 2b), making AnV-APC a more suitable probe to determine the effects of EGCG on platelet PS exposure.

### EGCG does not affect A23187-stimulated AnV-APC binding

We repeated our initial experiments but with AnV-APC. Washed platelets were treated with EGCG, the series of related catechins, or vehicle as control, prior to stimulation with A23187. Since in this experiment we were most interested in whether platelet PS exposure would be affected by the catechin polyphenols, event acquisition was triggered using forward scatter. (This is discussed further, below. EVs are too small to be readily resolved when acquisition is triggered using forward scatter.) We observed no significant inhibition of AnV-APC binding by any of the catechin polyphenols (Fig. 2c,d). These data suggest that EGCG and related catechin polyphenols do not directly inhibit phospholipid scrambling in platelets.

### EGCG does not inhibit scrambling of NBD-labelled PS

**Figure 3:**
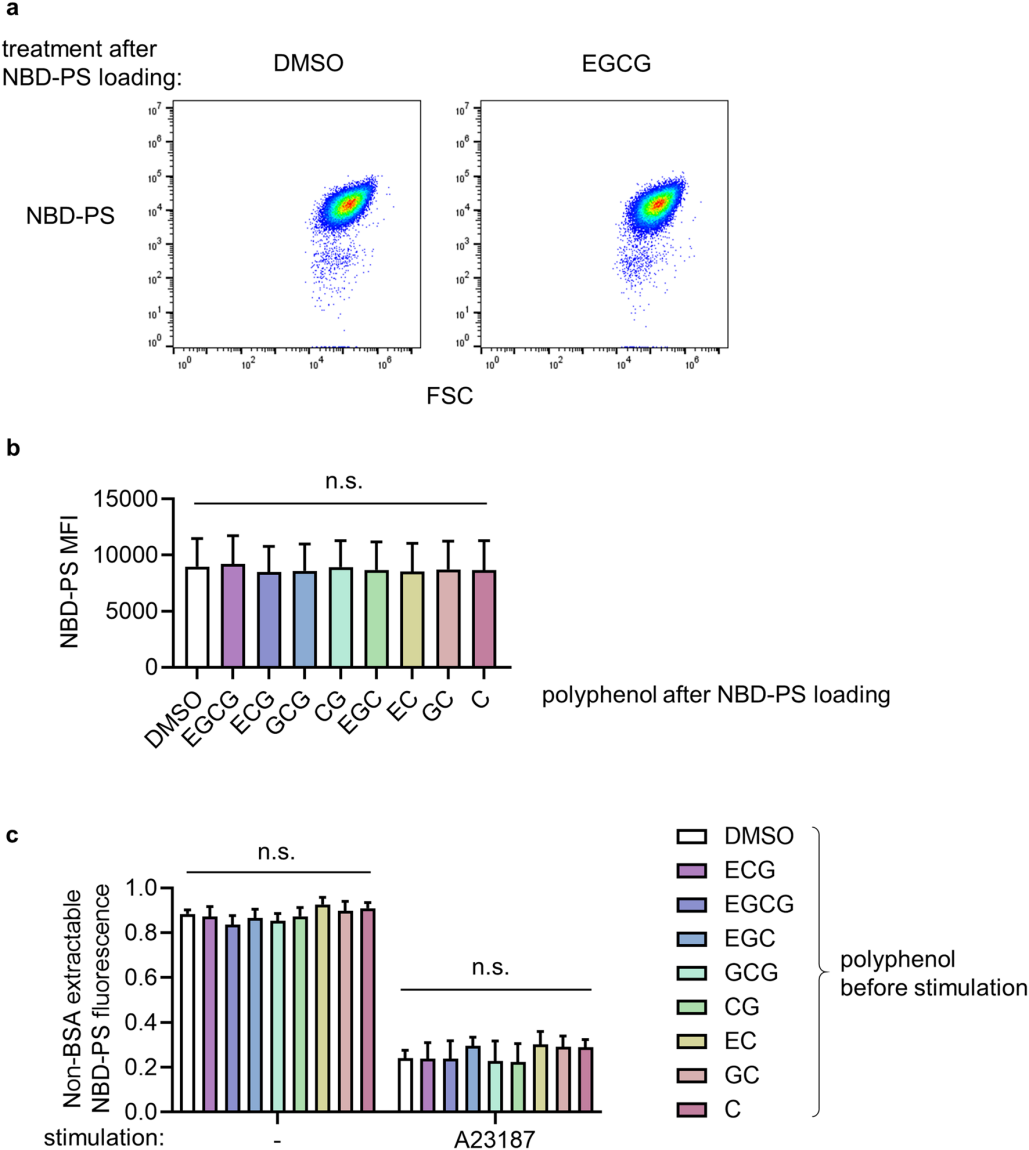
EGCG and related catechin polyphenols do not inhibit scrambling of NBD-labelled PS. Platelets were loaded with NBD-PS (0.5 μM, 45 min), fixed, then treated with the indicated catechin polyphenols (or DMSO as vehicle control). Representative density plots are shown in (**a**) and the MFI is shown in (**b**) (mean ± s.e.; n = 3; n.s. not significant). In (**a**), platelets were loaded with NBD-PS, treated with the indicated polyphenol, after which some samples were stimulated with A23187 as indicated. For each sample, NBD-PS fluorescence of platelets was determined before and after extraction of outer leaflet NBD-PS by BSA. Shown is the non BSA-extractable fluorescence as a proportion of the total fluorescence (mean ± s.e.; n = 4; n.s. not significant).

To confirm this conclusion through a complementary approach, we monitored the distribution of nitrobenzoxadiazole (NBD)-labelled PS. Washed platelets were loaded with NBD-PS, fixed, then treated with the catechin polyphenols. These drugs had no effect on NBD-PS fluorescence, indicating that they do not quench NBD-PS (Fig. 3a). NBD-PS in the outer leaflet can be extracted by bovine serum albumin (BSA) whereas NBD-PS in the inner leaflet cannot^33^. Washed platelets were incubated with NBD-PS, during which time almost all the NBD-PS internalised to the inner leaflet, as shown by the lack of extraction by subsequent addition of BSA to unstimulated platelets (Fig. 3b). In contrast, following stimulation with A23187, most of the NBD-PS could be extracted by BSA, demonstrating that it had moved the outer leaflet (Fig. 3b). The extent of NBS-PS extraction by BSA from stimulated platelets was not significantly different between catechin polyphenol-treated and vehicle-treated platelets, indicating that these catechins do not affect the extent of PS movement from the inner leaflet to the outer leaflet. This confirms that EGCG and related catechin polyphenols do not directly inhibit phospholipid scrambling in platelets.

### EGCG inhibits release of CD41^+^ EVs

**Figure 4:**
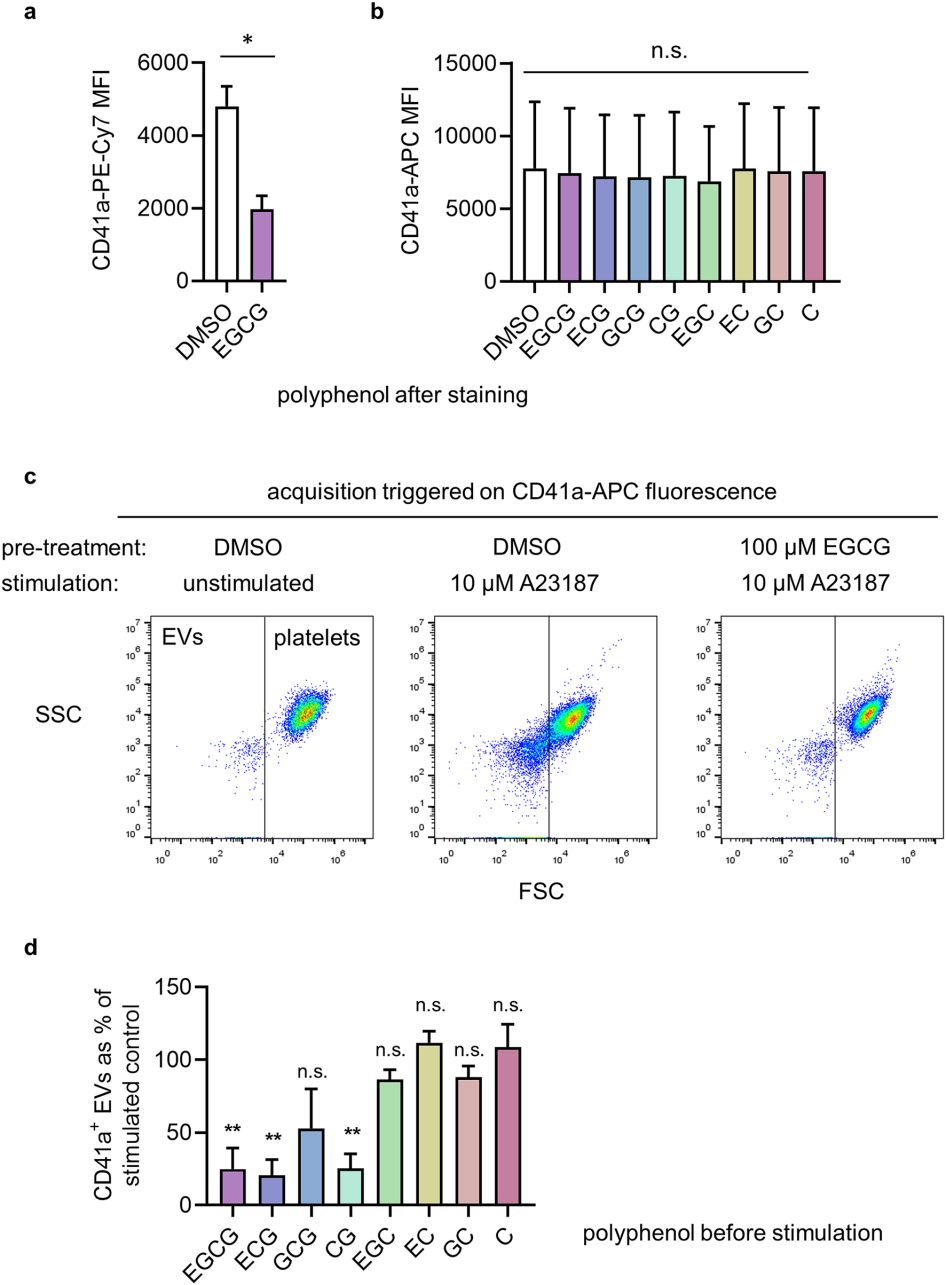
EGCG inhibits release of CD41a + EVs. In (**a**), platelets were stained with CD41a-PE-Cy7, fixed, then treated with EGCG; in (**b**), platelets stained with CD41a-APC, fixed, then treated with the indicated catechin polyphenol (or DMSO as vehicle control). The MFI is shown (mean ± s.e.; n = 4). In (**c**,**d**), platelets were treated with EGCG (or other catechin polyphenols in (**d**) or DMSO before stimulation with A23817. Note that AnV was not used in this experiment. APC fluorescence was used to trigger acquisition of CD41a^+^ events. The representative density plots therefore show FSC against side scatter (SSC). The number of CD41a^+^ EVs (< 1 μm) are shown in d as a percentage of EVs detected in DMSO (control)-treated, A23187-stimulated samples (mean ± s.e.; n = 4; **p < 0.01; n.s. not significant).

In our initial experiments we observed that EGCG appeared to inhibit the release of PS-exposing EVs. Event acquisition was triggered above a threshold set on FL3 fluorescence, using anti-CD41a-PE-Cy7. However, as with AnV-FITC, we found that EGCG partially quenched this fluorescence (Fig. 4a). Since EVs are smaller than intact platelets they have lower anti-CD41a-PE-Cy7 binding. When this fluorescence was partially quenched by EGCG, the fluorescence of EVs may have been too low to trigger event acquisition, making it appear that the number of EVs was reduced. In contrast, anti-CD41a-APC fluorescence was not quenched by EGCG or the related catechin polyphenols (Fig. 4b). This means that anti-CD41a-APC fluorescence (detected in FL4) could be used to trigger event acquisition, although we could not simultaneously use AnV-APC.

To determine whether EGCG or related catechin polyphenols affect EV release, washed platelets were treated with EGCG or related catechin polyphenols, or vehicle as control, prior to stimulation with A23187, and subsequently stained with anti-CD41a-APC. As shown in Fig. 4c,d, fewer CD41a^+^ EVs were detected following EGCG treatment compared to control. Moreover, EV release was also inhibited by two other catechin polyphenols that contained a gallate group (R2 in Supplmentary Table 1), namely epicatechin gallate and catechin gallate, but not by those without the gallate group (Fig. 4c,d).

## Discussion

Platelet PS exposure is a major contributor to thrombosis yet this pathway has been largely overlooked as a source of potential anti-thrombotic targets. TMEM16F, the major phospholipid scramblase in platelets^16^, is one such potential target. There are a few reported inhibitors that could be used as a starting point for developing a therapeutic, including R5421, niclosamide and EGCG. However, we have previously shown that R5421, a reported scramblase inhibitor, has substantial off-target effects in platelets^34^. Although niclosamide is worth future investigation, this anthelmintic drug also inhibits oxidative phosphorylation in target tapeworms^35^. EGCG is the major catechin polyphenol in green tea and has been associated with a wide range of health benefits, but also with a wide range of inhibitory effects on platelets and other cells. Moreover, a recent study proposed that EGCG does not inhibit TMEM16F exogenously expressed in cell lines, but rather that previously reported effects were due to EGCG’s quench of fluorophores coupled to annexin V. In this study, we investigated whether EGCG inhibits PS exposure catalysed by endogenous TMEM16F in human platelets.

Our results are consistent with Le et al.^31^, demonstrating that EGCG does not directly inhibit phospholipid scrambling in platelets. Phospholipid scrambling is triggered by a large rise in cytosolic Ca^2+^ concentration ([Ca^2+^]_cyt_). For this study we stimulated platelets with the Ca^2+^ ionophore, A23187. Platelet procoagulant activity can be stimulated by more physiological activators, such as a combination of thrombin and collagen^36–38^, which triggers a large rise in [Ca^2+^]_cyt_ in a subpopulation of platelets^11,13^. However, since EGCG may inhibit several intracellular signalling pathways in platelets^26,29,39^, we chose to bypass receptor signalling and directly increase [Ca^2+^]_cyt_ with A23187. Platelet PS exposure was monitored by binding of fluorophore-conjugated annexin V. Although EGCG appeared to reduce the extent of annexin V-FITC binding when platelets were treated with EGCG before stimulation, EGCG also reduced annexin V-FITC fluorescence when applied to platelets after annexin V staining and fixation. This indicates that the decrease in annexin V-FITC fluorescence is likely to be due to fluorescence quench by EGCG, not reduced annexin V binding. To test this conclusion, we used APC-conjugated annexin V, which we found was not quenched by EGCG under these conditions. Notably, EGCG did not inhibit the extent of annexin V-APC binding.

We considered the possibility that annexin V binding is not sufficiently sensitive to detect a small decrease in PS exposure due to partial inhibition of TMEM16F. On a PS/Ca^2+^ surface, Annexin V assembles into trimers and larger oligomers^40^, leading to a very steep relationship between % PS and annexin V binding in liposomes^41,42^. To investigate phospholipid scrambling through an alternative approach we used NBD-labelled PS. NBD-PS inserts into the outer leaflet of the plasma membrane as it is moved to the inner leaflet by flippase activity. EGCG did not quench the fluorescence of NBD-PS loaded into platelets. As shown in Fig. 3, EGCG also did not affect the scrambling of NBD-PS. Together, these data demonstrate that EGCG does not inhibit phospholipid scrambling and PS exposure in human platelets. EGCG therefore cannot be used as a template for the development of TMEM16F inhibitory anti-thrombotic drugs.

Unexpectedly, however, we found that EGCG inhibits the release of CD41a^+^ EVs from platelets. To detect EVs by flow cytometry, event acquisition was triggered by fluorescence rather than forward scatter. In our initial experiments (Fig. 1), event acquisition was triggered by PE-Cy7 conjugated to anti-CD41a. Since EGCG also quenches PE-Cy7 fluorescence, this may artificially reduce the number of acquired events and appear to reduce the number of EVs. However, anti-CD41a-APC fluorescence was not quenched under our experimental conditions, allowing us to use this antibody conjugate to trigger event acquisition. Despite no effect on PS exposure, EGCG inhibited EV release. An alternative explanation for our data is that EGCG caused the released EVs to be much smaller in size, such that they fell below the detection limit of our flow cytometry, as the Accuri C6 used here is poorly sensitive at detecting smaller EVs^43^. Whether EGCG reduces the number or the size of released EVs, our data show that EGCG disrupts platelet EV release independently of PS exposure. Moreover, this inhibition was shared by related gallate-containing catechin polyphenols, catechin gallate and epicatechin gallate, but not by catechin polyphenols that lack the gallate group. How EGCG inhibits EV release is not yet clear. The mechanisms underlying EV release from platelets are not well understood^44^, but analysis of unexpected pharmacological effects could reveal new pathways and new targets for anti-thrombotic therapies.

## Methods

### Washed platelet preparation

Washed platelets were prepared as previously described^34^. Blood was taken by venepuncture from healthy, drug-free volunteers who had given informed, written consent in accordance with the Declaration of Helsinki and with approval from the Human Biology Research Ethics Committee, University of Cambridge. Volunteers were aged between 20 and 60 years. Both male and female volunteers contributed to this study, and no difference in the response to EGCG was noted between platelets from male or female volunteers. Volunteers reported that they had taken neither aspirin, ibuprofen, paracetamol or antihistamine drugs in the period 10 days prior to donation, nor were taking any prescription medications. Blood was drawn into sodium citrate (3.2% v/v) vacutainers. Acid citrate dextrose (in mM: 85 tri-sodium citrate, 71 citric acid, 111 D-glucose) was added (1:7 v/v) and platelet rich plasma (PRP) was separated by centrifugation (200 g, 10 min, ambient temperature and without brake). PRP was collected and diluted 1:1 v/v with HEPES-buffered saline (HBS; in mM: 135 NaCl, 3 KCl, 10 HEPES, 1 MgCl_2_.6H_2_O, 0.34 Na_2_HPO_4_, 12 NaHCO_3_; pH 7.4) supplemented with 5 mM D-glucose (HBS-glucose). 100 nM prostaglandin E_1_ and apyrase (grade VII; 0.02 U/ml) were added to prevent platelet activation. Platelets were pelleted by centrifugation (600 g, 10 min, ambient temperature). The platelet pellet was resuspended in HBS-glucose, counted on a BD Accuri C6 flow cytometer and then diluted to 5 × 10^7^ platelets per ml unless otherwise specified. Apyrase was added (0.02 U/ml) and platelets were rested in a 30 °C water bath for 30 min before use. Prior to use, CaCl_2_ was added so stimulations occurred at 2 mM Ca^2+^.

### Flow cytometry—Annexin V binding

Platelets were pre-treated with 100 μM EGCG, related catechin polyphenols or 0.1% dimethylsulfoxide (DMSO) as a vehicle control for 10 min at room temperature. Platelets were stimulated with 10 μM A23817 for 10 min at room temperature. Following stimulations, platelets were stained fluorescently conjugated antibodies or proteins for 2 min at ambient temperature, then fixed with 1% paraformaldehyde (PFA) 1:5 v/v for 5 min and then diluted 1:2 v/vwith HBS for analysis by flow cytometry (BD Accuri C6). Antibodies used were PE-Cy7-conjugated anti-CD41a antibody (1:100; eBioscience 25-0419-42) or APC-conjugated anti-CD41a antibody (1:100; eBioscience 17-0419-42) to positively identify platelets. Annexin V (AV)-FITC (1:100 in HBS-glucose with 2.5 mM CaCl_2_) or AV-APC (1:20 in HBS-glucose with 2.5 mM CaCl_2_; BioLegend 640941) were used to detect surface PS exposure. EV were gated from platelets based on FSC profile. The forward scatter and side scatter profiles (FSC/SSC) of the platelets were typical for healthy donors.

### Scramblase Activity Assay

Platelets were loaded with 0.5 µM NBD-PS for 45 min in a 30 °C water bath. Platelets were then treated with 100 μM EGCG, related catechin polyphenols or 0.1% DMSO for 10 min at room temperature. Platelets were stimulated with 10 μM A23817 for 10 min at room temperature in the presence of 2 mM CaCl_2_. After 10 min, platelets were transferred to HBS + /- BSA for 5 min to extract NBD-PS in the outer leaflet of the plasma membrane. Platelets were then fixed in 1% PFA before being analysed by flow cytometry.

### Data analysis and statistics

Flow cytometry density plots were generated in FlowJo v10.7 (https://www.flowjo.com/). As described previously^34^, the number of biological repeats in each experiment is detailed in figure legends and represents independent platelet preparations from different donors. Statistical analyses were performed in GraphPad Prism v.9 (https://www.graphpad.com/). All statistical tests were carried out using matched analyses of independent values (not technical replicates). Where individual treatment conditions were compared to DMSO controls, two-tailed Student’s paired t-tests were used. Where multiple treatment conditions were compared to DMSO controls, data were analysed using one-way repeated measures ANOVA and Dunnett’s multiple comparison tests as appropriate (and only when F in ANOVA achieved statistical significance). *p* values < 0.05 were taken to be statistically significant. *p < 0.05, **p < 0.01, ***p < 0.001.

### Materials

Unless otherwise stated, the reagents used in this study were from Sigma Aldrich (Poole, Dorset, UK). Sodium citrate-containing vacutainers were from Greiner Bio-One. Paraformaldehyde solution was from Alfa Aesar. Epigallocatechin gallate (EGCG), epicatechin gallate (ECG), catechin gallate (CG), epicatechin (EC), catechin (C), gallocatechin gallate (GCG), epigallocatechin (EGC) and gallocatechin (GC) were from Cayman Chemicals (Michigan, USA).

## Supplementary Information

Supplementary Information 1.
