# Supplementary figures and images for: Epigallocatechin gallate inhibits release of extracellular vesicles from platelets without inhibiting phosphatidylserine exposure
Source: Sci Rep. 2021 Sep 3;11:17678. doi: 10.1038/s41598-021-97212-8 (PMC8417220; doi:10.1038/s41598-021-97212-8)

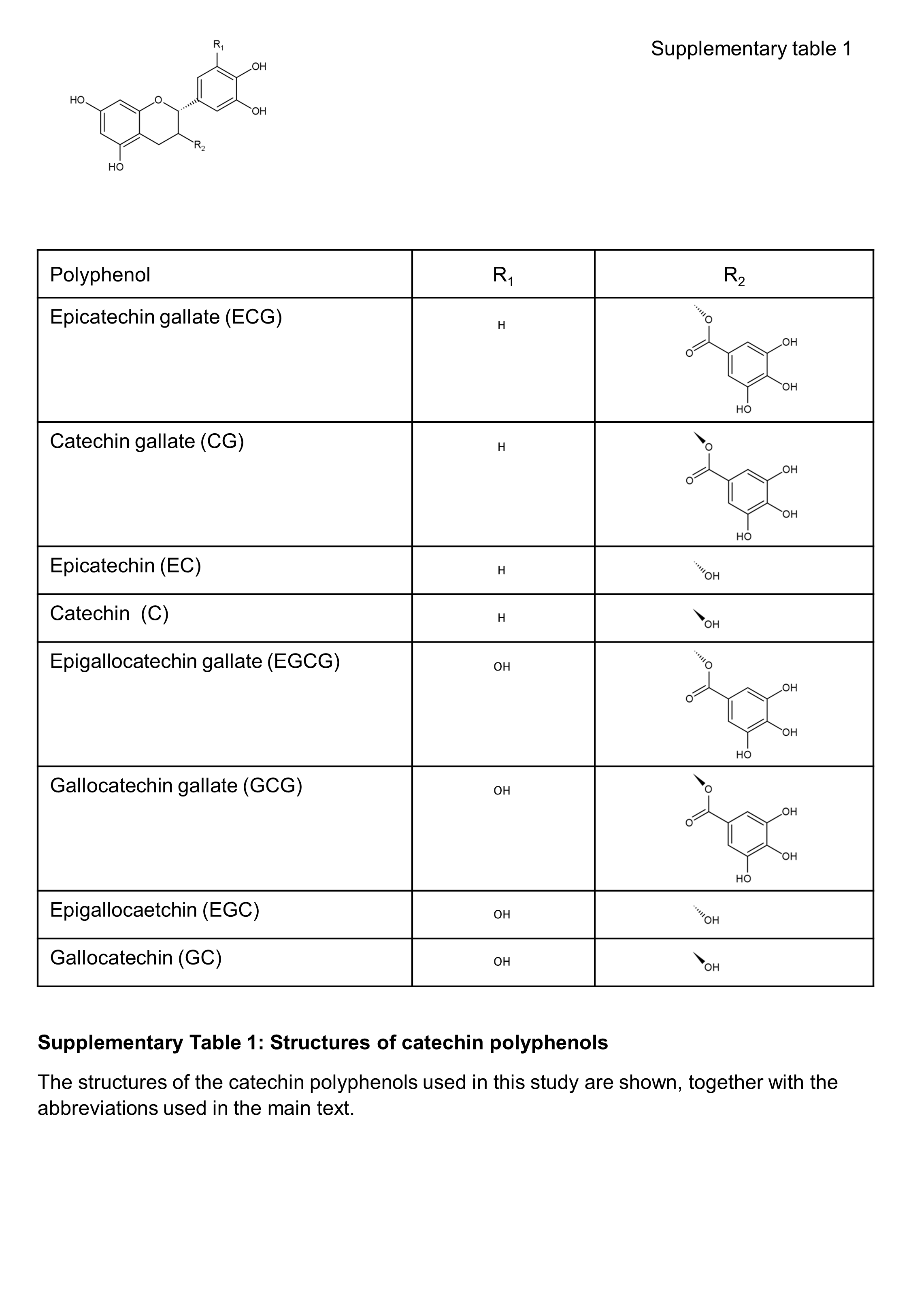

Supplement: Supplementary file 1 — Supplementary Information 1. [file 41598_2021_97212_MOESM1_ESM.tif]
